# Mice deficient in the *Shmt2* gene have mitochondrial respiration defects and are embryonic lethal

**DOI:** 10.1038/s41598-017-18828-3

**Published:** 2018-01-11

**Authors:** Haruna Tani, Sakiko Ohnishi, Hiroshi Shitara, Takayuki Mito, Midori Yamaguchi, Hiromichi Yonekawa, Osamu Hashizume, Kaori Ishikawa, Kazuto Nakada, Jun-Ichi Hayashi

**Affiliations:** 1 Graduate School of Life and Environmental Sciences, University of Tsukuba, 1-1-1 Tennodai, Tsukuba, Ibaraki 305-8572 Japan; 2 Laboratory for Transgenic Technology, Tokyo Metropolitan Institute of Medical Science, 2-1-6 Kamikitazawa, Setagaya-ku, Tokyo 156-8506 Japan; 3 Faculty of Life and Environmental Sciences, University of Tsukuba, 1-1-1 Tennodai, Tsukuba, Ibaraki 305-8572 Japan; 4 University of Tsukuba, 1-1-1 Tennodai, Tsukuba, Ibaraki 305-8572 Japan

## Abstract

Accumulation of somatic mutations in mitochondrial DNA (mtDNA) has been proposed to be responsible for human aging and age-associated mitochondrial respiration defects. However, our previous findings suggested an alternative hypothesis of human aging—that epigenetic changes but not mutations regulate age-associated mitochondrial respiration defects, and that epigenetic downregulation of nuclear-coded genes responsible for mitochondrial translation [e.g., *glycine C-acetyltransferase* (*GCAT*), *serine hydroxymethyltransferase 2* (*SHMT2*)] is related to age-associated respiration defects. To examine our hypothesis, here we generated mice deficient in *Gcat* or *Shmt2* and investigated whether they have respiration defects and premature aging phenotypes. *Gcat*-deficient mice showed no macroscopic abnormalities including premature aging phenotypes for up to 9 months after birth. In contrast, *Shmt2*-deficient mice showed embryonic lethality after 13.5 days post coitum (dpc), and fibroblasts obtained from 12.5-dpc *Shmt2*-deficient embryos had respiration defects and retardation of cell growth. Because *Shmt2* substantially controls production of N-formylmethionine-tRNA (fMet-tRNA) in mitochondria, its suppression would reduce mitochondrial translation, resulting in expression of the respiration defects in fibroblasts from *Shmt2*-deficient embryos. These findings support our hypothesis that age-associated respiration defects in fibroblasts of elderly humans are caused not by mtDNA mutations but by epigenetic regulation of nuclear genes including *SHMT2*.

## Introduction

Because mitochondria produce reactive oxygen species endogenously and preferentially accumulate exogenous chemical carcinogens, mitochondrial DNA (mtDNA) is exposed to these mutagens, resulting in accumulation of somatic mutations with age^1–5^. Some of these somatic mutations in human mtDNA are pathogenic, because the same mutations are found in patients with mitochondrial diseases caused by mitochondrial respiration defects. Therefore, the mitochondrial theory of aging^1–5^ proposes that accumulation of somatic mutations in mtDNA is responsible for human aging and age-associated mitochondrial respiration defects.

However, it is also possible that abnormalities in nuclear DNA but not in mtDNA induce age-associated mitochondrial respiration defects, because both nuclear DNA and mtDNA encode proteins required for mitochondrial respiratory function^1^. To determine which genome, nuclear or mitochondrial, is responsible for the respiration defects in the fibroblasts of elderly humans, we previously carried out intercellular transfer of mtDNA^6^ or nuclear DNA^7^ by using mtDNA-less HeLa cells^8^; the results led us to propose that nuclear recessive mutations induce the age-associated respiration defects. In contrast, the mitochondrial theory of aging has been supported by studies of mtDNA mutator mice^9,10^, which were generated by introducing a proofreading-deficient mtDNA polymerase gene. These mice showed accelerated accumulation of somatic mutations in mtDNA, resulting in accelerated expression of respiration defects and premature aging phenotypes^9,10^.

Therefore, it has been controversial whether human aging and age-associated respiration defects are controlled by the accumulation of somatic mutations in mtDNA^9,10^ or by nuclear recessive mutations^7^. More recently, epigenetic regulation of cellular senescence has been proposed in human fibroblasts^11^. Our recent study^12^ addressed these issues by deep sequencing analysis of mtDNA and showed that mtDNA in fibroblasts from elderly humans does not accumulate somatic mutations. Moreover, reprogramming of these fibroblasts by generating induced pluripotent stem cells (iPSCs) restores normal respiratory function^12^. This led us to hypothesise that age-associated respiration defects are controlled not by mutations in either nuclear or mtDNA, but by epigenetic regulation of nuclear genes. Our microarray screening results suggest that epigenetic downregulation of the nuclear genes *glycine C-acetyltransferase* (*GCAT*) and *serine hydroxymethyltransferase 2* (*SHMT2*) is involved in age-associated respiration defects of the fibroblasts of elderly humans^12^. Because the products of both genes are localized in mitochondria and regulate glycine production in mitochondria^13,14^, their downregulation would induce defects in mitochondrial translation and respiratory function, resulting in the age-associated respiration defects found in the fibroblasts of elderly humans^6,7^. To examine this possibility, we generated mice deficient in *Gcat* or *Shmt2*, and investigated whether these mice would have mitochondrial respiration defects and premature aging phenotypes.

## Results

### Generation of mice deficient in the *Gcat* or *Shmt2* genes

We generated knockout mouse strains deficient in the *Gcat* gene or the *Shmt2* gene by using the CRISPR/Cas9 system. Target sequences were designed according to the mouse *Gcat* and *Shmt2* sequences (Supplementary Fig. S1). Cas9 mRNA and single-guide RNAs (sgRNAs) were synthesized as reported previously^15^, and were microinjected into fertilized eggs (pronuclear stage) from C57BL/6J (hereafter referred to as B6J) mice. The microinjected eggs were transferred to the oviducts of pseudo-pregnant females.

**Figure 1:**
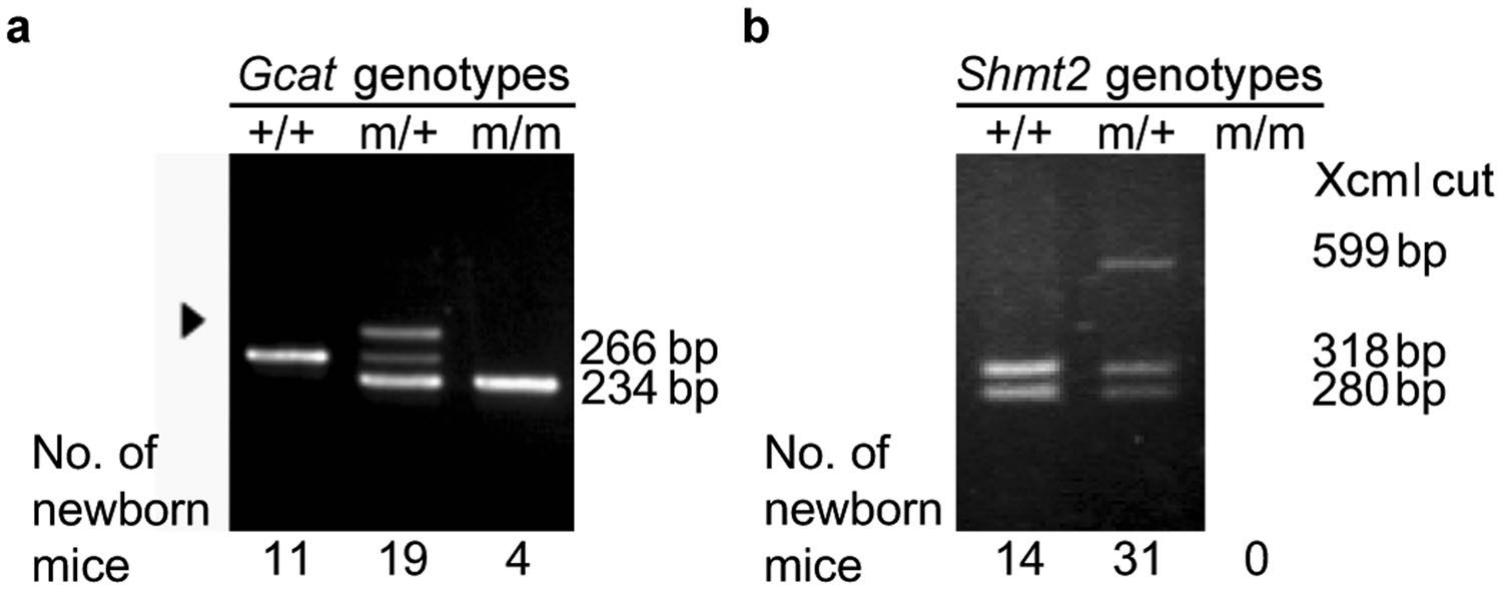
Genotyping of F_1_ pups obtained by mating heterozygous females and males. (**a**) Mutations in *Gcat*. PCR products of 266 bp and 234 bp correspond to *Gcat* without mutations (+/+) and with mutations (m/m; see Supplementary Fig. S1 for sequences), respectively. The presence of both fragments indicates heterozygosity (m/+). Arrowhead shows an additional fragment that may represent heteroduplex molecules. (**b**) Mutation in *Shmt2*. Because an XcmI site was eliminated by an insertion of T (Supplementary Fig. S1), XcmI digestion of PCR products without the mutation produces two fragments (318 bp and 280 bp), whereas digestion of PCR products with the T insertion produces a single fragment (599 bp including the T insertion). The presence of all three fragments indicates heterozygosity (m/+). No mice with homozygous mutations (m/m) were found, indicating their embryonic lethality.

In the case of *Gcat* knockout mice, 41 of 70 mice were mutation-positive in the Surveyor assay (see Methods). We analysed the sequence around the target region in the mice with mutations and selected one male mouse with an insertion and a deletion that would disrupt *Gcat* gene function (Supplementary Fig. S1); we used this mouse as a founder for further breeding to obtain heterozygous (*Gcat* m/+) females and males. By mating heterozygous females with heterozygous males, we obtained 34 pups. Genotyping showed that 11 pups had no mutation, 19 were heterozygous, and 4 were homozygous (*Gcat* m/m) (Fig. 1a). We then obtained offspring (*Gcat*+/+, m/+, m/m) by *in vitro* fertilization using heterozygous females and a heterozygous male.

In the case of *Shmt2* knockout mice, 20 of 25 mice were mutation-positive in the Surveyor assay. We selected one female mouse with a single-nucleotide insertion (T) resulting in a frame shift that would disrupt *Shmt2* gene function (Supplementary Fig. S1) and used this mouse as a founder for further breeding to obtain heterozygous (*Shmt2* m/+) females and males. By mating heterozygous females with heterozygous males, we obtained 45 pups. Genotyping by Xcm I digestion of the PCR products showed that 14 mice had no mutation and 31 were heterozygous, but no mice had a homozygous mutation (Fig. 1b), indicating the lethality of embryos with a homozygous mutation in *Shmt2* (*Shmt2* m/m).

### Characterization of mice deficient in the *Gcat* gene

**Figure 2:**
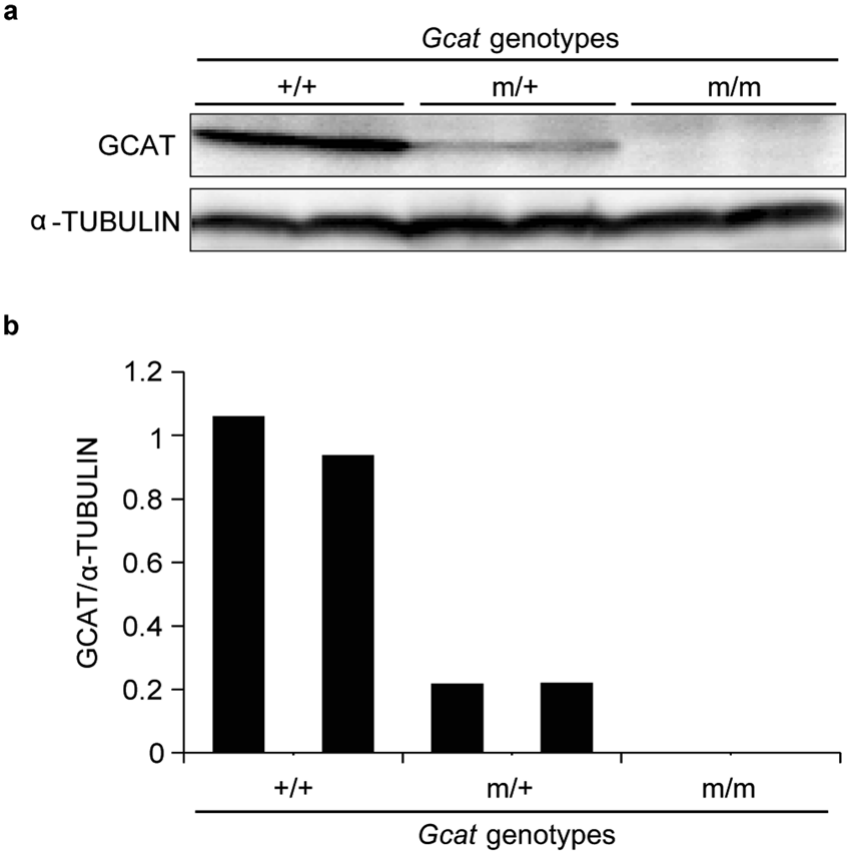
Suppression of GCAT protein production in 5-month-old *Gcat* m/m mice. (**a**) Western blot analysis of the GCAT protein in the livers of mice of the indicated genotypes. (**b**) Quantification of Western blot data.

Because the *Gcat* m/m mice did not show embryonic lethality, we performed Western blot analysis to confirm the suppression of the *Gcat* gene in these mice. The GCAT protein was not detectable in livers of 5-month-old *Gcat* m/m mice, but was detectable in wild-type (+/+) or heterozygous (m/+) mice (Fig. 2). These observations confirm complete absence of the GCAT protein in *Gcat* m/m mice.

However, no growth retardation or obvious macroscopic abnormalities including premature aging phenotypes, such as hair greying, alopecia, or kyphosis, were observed in *Gcat* m/m mice for at least 9 months after birth, even though they did not have the GCAT protein. Mice with a heterozygous mutation in the *Shmt2* gene (*Shmt2* m/+ mice) also showed no growth retardation or no obvious macroscopic abnormalities for at least 9 months after birth. Further investigations would be required to determine whether these mice have any premature aging phenotypes or shorter lifespan than those of mice without the mutations.

### Characterization of embryos deficient in the *Shmt2* gene

**Figure 3:**
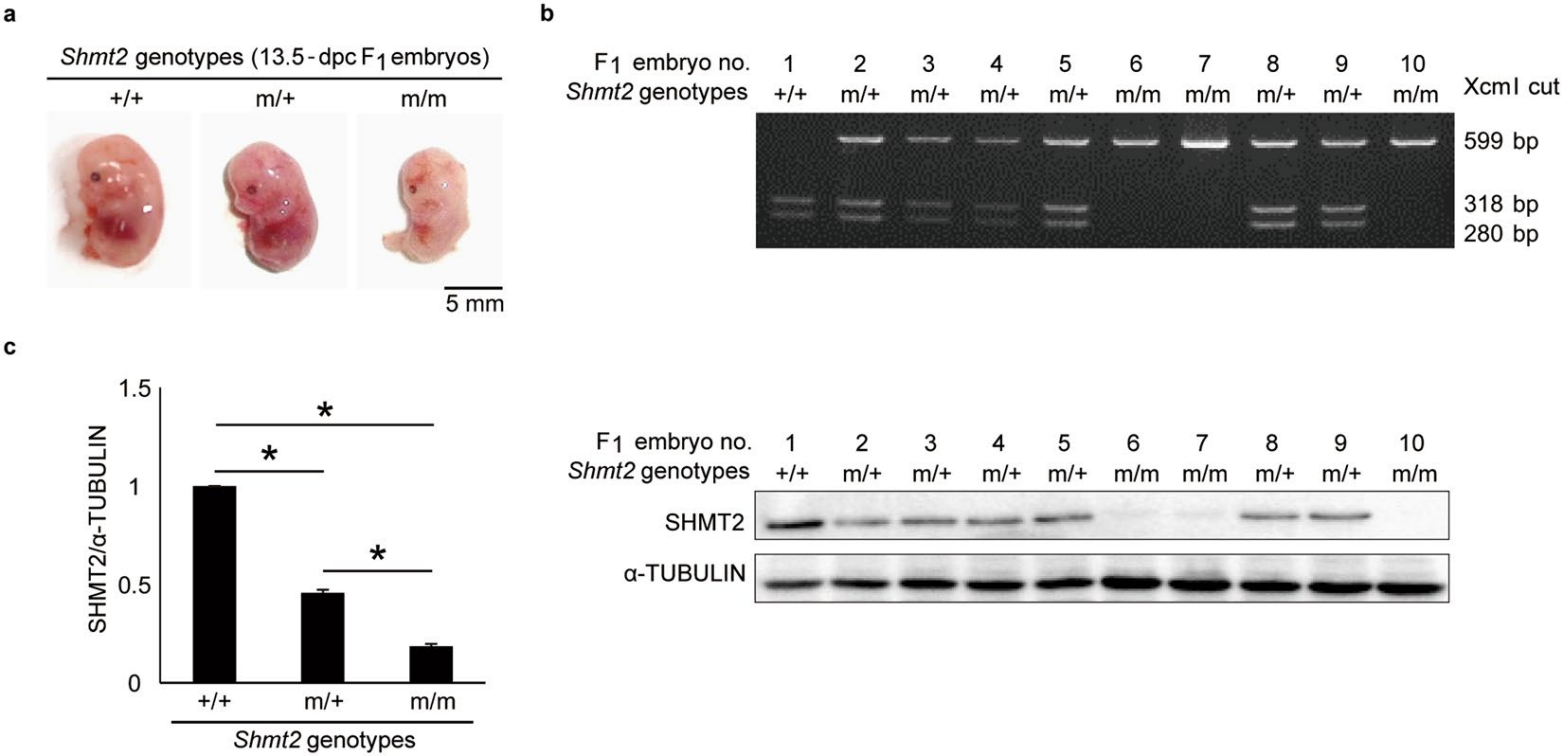
Lethality of 13.5-dpc F_1_ embryos with a homozygous mutation in *Shmt2* obtained by mating heterozygous females and males. (**a**) Macroscopic abnormalities. (**b**) Genotyping of the *Shmt2* mutation. (**c**) Examination of the presence of the SHMT2 protein by Western blot analysis. Experiments were performed in triplicate. Data are means ± s.e.m. *P < 0.05, ANOVA followed by Tukey’s multiple comparison test.

The absence of *Shmt2* m/m mice among the offspring (Fig. 1b) suggested their embryonic lethality. To investigate the embryonic lethality, we used 12.5-dpc (days post coitum) and 13.5-dpc F_1_ embryos obtained by mating heterozygous females with heterozygous males. We found no macroscopic abnormalities in any of the 12.5-dpc embryos, but detected abnormalities such as small size and anaemia in three of the ten 13.5-dpc embryos (Fig. 3a), indicating that the lethality occurs after13.5 dpc.

Then, we genotyped the embryos by Xcm I digestion of PCR products. Of the ten 13.5-dpc embryos, one had no mutation, six were heterozygous, and three had homozygous mutations (Fig. 3b); the three embryos with homozygous mutations were the same as those showing macroscopic abnormalities. Western blot analysis (Fig. 3c) showed that the amounts of SHMT2 protein in embryos without the mutation were about twice those in heterozygous embryos. In embryos with homozygous mutations, low amounts of the SHMT2 protein were detectable (Fig. 3c), probably due to contaminating maternal cells, such as placenta or blood cells.

### Examination of *Gcat* and *Shmt2* expression during embryonic stages

**Figure 4:**
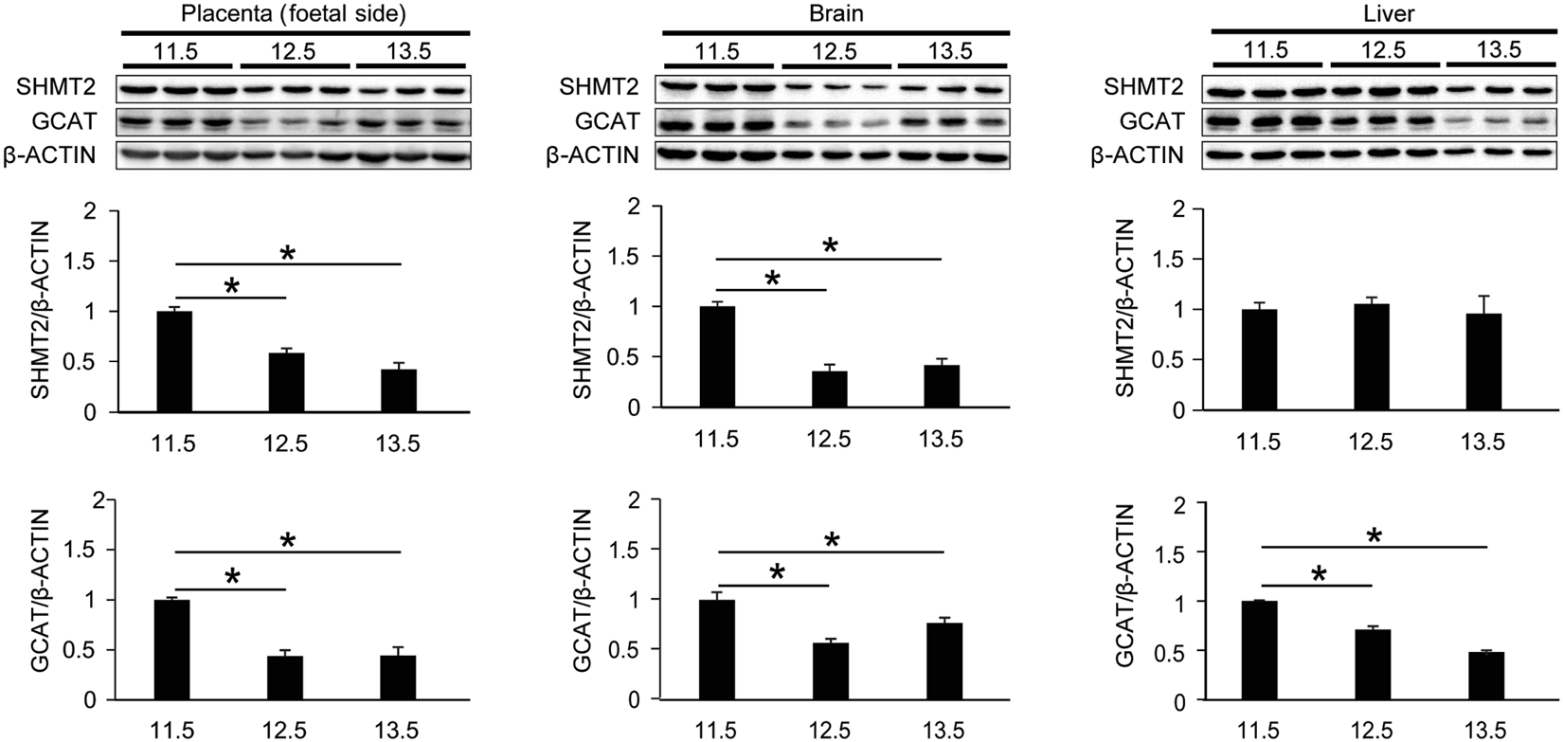
Examination of SHMT2 and GCAT protein levels in the placenta (foetal side), brain, and liver of 11.5-, 12.5- and 13.5-dpc wild-type B6J embryos by Western blot analysis. Experiments were performed in triplicate. Data are means ± s.e.m. *P < 0.05, ANOVA followed by Tukey’s multiple comparison test.

A question that arises from the absence of embryonic lethality in *Gcat* m/m mice (Fig. 1a) is whether the expression of the *Gcat* gene is not required at any embryonic stage. To answer this question, we examined the levels of the GCAT and SHMT2 proteins in the placenta (foetal side), brain, and liver from 11.5-, 12.5- and 13.5-dpc embryos without mutations by Western blot analysis. Both proteins were present in all three tissues at all embryonic stages examined; their levels decreased slightly but significantly after 11.5 dpc, except that the level of SHMT2 in the liver did not change significantly (Fig. 4). Therefore, the absence of embryonic lethality in *Gcat* m/m mice cannot be explained by the absence of *Gcat* expression during embryogenesis.

### Isolation and characterization of mouse embryonic fibroblast lines from the embryos of *Shmt2* m/m mice

**Figure 5:**
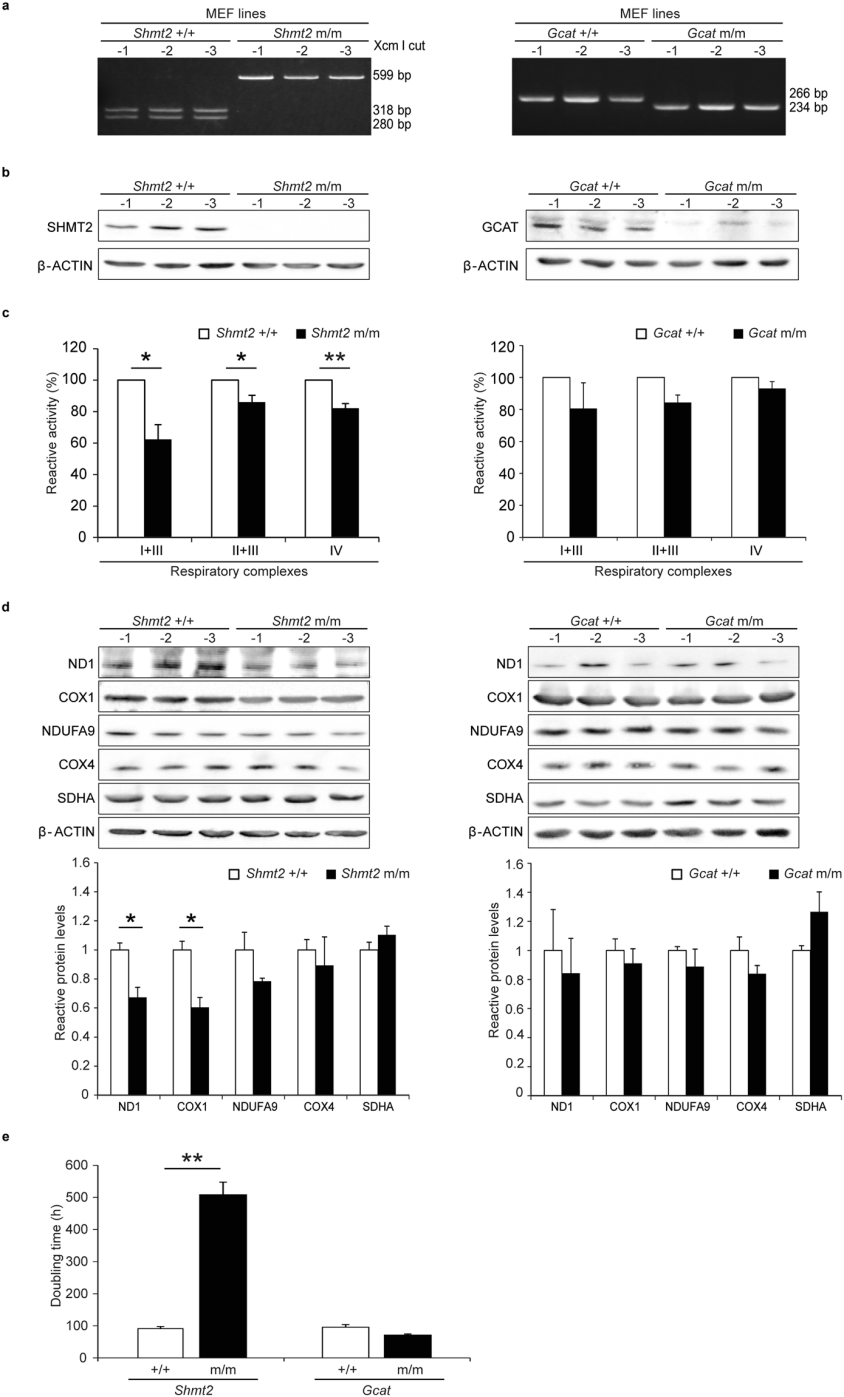
Characterization of MEF lines generated from 12.5-dpc embryos with no mutation (*Shmt2*+/+; *Gcat*+/+) and with homozygous mutations (*Shmt2* m/m; *Gcat* m/m). (**a**) Genotyping of the mutation. (**b**) Western blot analysis of SHMT2 protein and GCAT protein. (**c**) Biochemical analysis of relative enzymatic activities of mitochondrial respiratory complexes. (**d**) Western blot analysis of the subunits of mitochondrial respiratory complexes encoded by mtDNA (ND1, COX1) and nuclear DNA (NDUFA9, COX4, SDHA). (**e**) Doubling times in culture. Experiments were performed in triplicate. Data are means ± s.e.m. *P < 0.05, **P < 0.01, Student’s *t*-test.

To investigate the cause of embryonic lethality of *Shmt2* m/m mice, we obtained two pregnant mice by mating heterozygous mice, and isolated a mouse embryonic fibroblast (MEF) line from each of the 12.5-dpc embryos. Genotyping showed that three lines had no mutation (*Shmt2*+/+ MEF), five were heterozygous, and eight had homozygous mutations (*Shmt2* m/m MEF). We used three *Shmt2*+/+ MEF lines and three *Shmt2* m/m MEF lines for further investigation (Fig. 5a). The absence of the SHMT2 protein in the *Shmt2* m/m MEF lines was confirmed by Western blot analysis (Fig. 5b). Given that MEF lines do not contain maternal cells, the presence of low amounts of SHMT2 in 12.5-dpc *Shmt2* m/m embryos (Fig. 3c) was likely due to contamination with maternal cells.

Then, we examined whether the absence of SHMT2 results in mitochondrial respiration defects by using *Shmt2*+/+ MEFs and *Shmt2* m/m MEFs. First, we performed biochemical analysis of respiratory function, and found the reduced activities of mitochondrial respiratory complexes in *Shmt2* m/m MEFs in comparison with *Shmt2*+/+ MEFs (Fig. 5c). Next, we used Western blot analysis to compare the amounts of nuclear- and mtDNA-coded subunits of respiratory complexes between *Shmt2*+/+ MEFs and *Shmt2* m/m MEFs, and found preferential decrease of mtDNA-coded subunits in *Shmt2* m/m MEFs (Fig. 5d). Probably, the absence of SHMT2 (Fig. 5b) induced significant respiration defects (Fig. 5c) as a consequence of the reduction of one-carbon metabolism to produce glycine and N-formylmethionine-tRNA (fMet-tRNA)^13,14,16^, both of which are required for the mitochondrial translation that produces mtDNA-coded subunits of respiratory complexes.

Finally, we compared the doubling times between *Shmt2*+/+ MEFs and *Shmt2* m/m MEFs, and found significant growth retardation in *Shmt2* m/m MEFs (Fig. 5e). Therefore, the embryonic lethality observed in *Shmt2* m/m mice is likely due to both the respiration defects and growth retardation caused by the *Shmt2* deficiency.

### Isolation and characterization of MEF lines from the embryos of *Gcat* m/m mice

Next, we generated MEF lines from *Gcat* m/m embryos, and examined their respiratory function and doubling times to investigate why *Gcat* m/m embryos are not embryonic lethal. We obtained two pregnant mice by mating heterozygous mice, and isolated a MEF line from each of the 12.5-dpc embryos. Genotyping showed that seven lines had no mutation (*Gcat*+/+ MEF), seven were heterozygous, and three had homozygous mutations (*Gcat* m/m MEF). We randomly selected three of the seven *Gcat*+/+ MEF lines and the three *Gcat* m/m MEF lines for further investigation (Fig. 5a). The complete absence of the GCAT protein in *Gcat* m/m MEF lines was confirmed by Western blot analysis (Fig. 5b).

Comparison of respiratory function and doubling time between *Gcat*+/+ MEFs and *Gcat* m/m MEFs showed no respiration defects and no growth retardation in *Gcat* m/m MEFs (Fig. 5c and e). Moreover, the amounts of nuclear- and mtDNA-coded subunits of respiratory complexes did not differ substantially between *Gcat*+/+ MEF and *Gcat* m/m MEF lines (Fig. 5d). These observations suggest that the absence of respiration defects and growth retardation in *Gcat* m/m MEF lines are related to the absence of embryonic lethality of *Gcat* m/m mice.

## Discussion

In our previous studies^12,17^, we put forward the hypothesis that age-associated respiration defects in human fibroblasts are not due to mutations but to the epigenetic regulation, because reprogramming fibroblasts from elderly humans by generating iPSCs restores normal mitochondrial respiratory function. Our hypothesis also proposed that epigenetic downregulation of human *GCAT* or *SHMT2* or both would partly be related to age-associated respiration defects. To test this hypothesis, we generated mice deficient in *Gcat* or *Shmt2*, and examined whether suppression of these genes induces respiration defects.

Mice deficient in *Shmt2* (*Shmt2* m/m) showed embryonic lethality, but mice deficient in *Gcat* (*Gcat* m/m) did not (Fig. 1). To further investigate the embryonic lethality of *Shmt2* m/m mice, we isolated MEF lines from 12.5-dpc embryos and showed that *Shmt2* m/m MEF lines had respiration defects (Fig. 5c). Moreover, the absence of SHMT2 (Fig. 5b) reduced the amounts of the mtDNA-coded subunits of the respiratory complexes (Fig. 5d). Therefore, these results are consistent with our hypothesis^12,17^ that epigenetic downregulation of human *SHMT2* is involved in age-associated respiration defects. Probably, age-associated downregulation of human *SHMT2* ^12^ suppresses one-carbon metabolism to produce glycine and fMet-tRNA^13,14^, both of which are required for translation in mitochondria, and thereby decreases the production of the mtDNA-encoded subunits of the respiratory complexes, resulting in expression of age-associated respiration defects.

Questions that then arise are why *Gcat* m/m mice are not embryonic lethal (Fig. 1), and why *Gcat* m/m MEF lines do not show respiration defects (Fig. 5c), even though both *Gcat* and *Shmt2* genes are involved in glycine production in mitochondria^13,14^. This discrepancy could be resolved by assuming that embryonic lethality and the respiration defects induced by *Shmt2* disruption result from fMet-tRNA depletion but not glycine depletion. The SHMT2 pathway generates fMet-tRNA via two processes; one is conversion of serine and tetrahydrofolate (THF) to glycine and 5,10-methylene-THF, and the other is conversion of the resultant glycine to 5,10-methylene-THF via the glycine cleavage system (GCS)^14^. In contrast, the GCAT pathway, which involves the *L-threonine dehydrogenase* (*Tdh*) gene, contributes to the generation of fMet-tRNA only via conversion of threonine to glycine followed by GCS to produce 5,10-methylene-THF^14^. Moreover, mouse embryonic stem cells use both SHMT2 and GCAT pathways for production of fMet-tRNA, but differentiated tissues use the SHMT2 pathway predominantly due to inactivation of the GCAT pathway by suppression of *Tdh* ^13^. Given that lethality of *Shmt2* m/m embryos occurs after 13.5 dpc (Fig. 3), it can be supposed that *Shmt2* m/m embryos could not produce sufficient amounts of fMet-tRNA after 13.5 dpc due to disruption of *Shmt2* and suppression of *Tdh*, resulting in respiration defects and embryonic lethality. In contrast, *Gcat* m/m embryos produce fMet-tRNA even after 13.5 dpc due to the active SHMT2 pathway, resulting in the absence of respiration defects and embryonic lethality. Furthermore, the results of an *in vitro* study^18^ suggest that SHMT2 catalyzes not only conversion of serine to glycine but also conversion of threonine to glycine, which indicates compensation of the GCAT pathway by SHMT2 pathway; this compensation could also explain the absence of respiration defects in *Gcat* m/m MEFs and the absence of lethality in *Gcat* m/m embryos.

In addition to the mitochondrial respiration defects, *Shmt2* m/m MEFs also showed significant growth retardation, but *Gcat* m/m MEFs showed neither respiration defects nor growth retardation (Fig. 5). The growth retardation of *Shmt2* m/m MEFs could be due to the respiration defects, because our previous studies^19,20^ provided evidence that respiration defects caused by pathogenic mtDNA mutations in mouse tumor cells delay their growth under the skin of syngenic B6J mice. However, it is also possible that growth retardation of *Shmt2* m/m MEF is due to impaired nucleotide production, because *Shmt2* also contributes to nucleotide production via one-carbon metabolism^13,14^, and plays an important role in growth or survival of tumor cells^21–23^ and immune cells^24^. All these observations suggest that *Shmt2* disruption impairs one-carbon metabolism producing nucleotides and fMet-tRNA^13,14^, and thus could be responsible for growth retardation and respiration defects, resulting in the lethality of *Shmt2* m/m embryos (Figs 1 b and 3a).

At the time of writing this report, the *Gcat* m/m mice and *Shmt2* m/+ mice (9 month-of-age) showed no macroscopic abnormalities including premature aging phenotypes, such as kyphosis, greying, or alopecia, which have been observed in the mtDNA mutator mice^9,10^. In a previous study^25^, we generated mtDNA mutator mice with the same B6J nuclear background as that of *Gcat* m/m or *Shmt2* m/+ mice, and found that their lifespan was short and they had kyphosis but no greying or alopecia. This premature aging phenotype (kyphosis) in our mtDNA mutator m/m mice was also observed in mito-miceΔ carrying mtDNA with a large-scale deletion and having the B6J nuclear background, which we generated previously as a model for mitochondrial diseases^26,27^. Given that median survival times of heterozygous and homozygous mtDNA mutator mice with the B6J nuclear background were 27 and 10 months, respectively^25^, further studies are required to examine whether *Gcat* m/m and *Shmt2* m/+ mice eventually have shorter lifespans than that of B6J mice and express a premature aging phenotype of kyphosis.

## Methods

### Ethics statement

All animal experiments were performed in accordance with protocols approved by the Institutional Animal Care and Use Committee of the University of Tsukuba, Japan (permit number: 15–313), and by the Animal Use and Care Committee of the Tokyo Metropolitan Institute of Medical Science (approval numbers: 14046, 15023, 16040).

### Mice

*Gcat*- or *Shmt2*- deficient mouse strains were generated by using the CRISPR/Cas9 system. Target sequences were designed according to the mouse *Gcat* and *Shmt2* sequences (GenBank accession nos. NM_001161712 and NM_001252316, respectively). Cas9 mRNA and sgRNAs were synthesized as reported previously^15^, and were microinjected into fertilized eggs (pronuclear stage) obtained from B6J mice (ARK Resource, Kumamoto, Japan).

### Surveyor assay

The Surveyor assay was used to detect mutations. Briefly, DNA samples from the tails of mice were prepared using a Maxwell 16 system (Promega, Madison, WI, USA). PCR was performed using a TaKaRa Ex Taq Hot Start Version (Takara Bio, Shiga, Japan) and the following primer sets: 5′-gtt gtt ctg att tgc tgc cg-3′ and 5′-tct ctc acc caa aag gtg ct-3′ for *Gcat*, and 5′-gag ttg acc aaa act gcc ct-3′ and 5′-tca agc ccc ata aac tgg tc-3′ for *Shmt2*. After amplification, the assay was performed using a Surveyor Mutation Detection Kit (Transgenomic, Omaha, NE, USA).

### Genotyping

PCR was performed with the same primer sets as in the Surveyor assay and an additional set for *Gcat* (5′-gat gtg ggc cag ctt cat-3′, 5′-gcc tcc att cac atg caa-3′). To genotype mice carrying the mutation in *Shmt2*, PCR samples were digested with XcmI.

### DNA sequencing

For sequence analysis of *Gcat* and *Shmt2* in mice and MEF lines, PCR amplifications were performed by using the same primer sets as used for genotyping. Amplified DNA fragments were directly purified or were recovered from gels after electrophoresis, and were directly sequenced (Supplementary Fig. S1).

### MEF lines and cell culture

MEF lines were derived from the skin of 12.5-dpc embryos and grown in minimum essential medium (Life Technologies) containing 10% foetal bovine serum (Sigma) and 1% penicillin/streptomycin (Nacalai Tesque, Kyoto, Japan).

### Biochemical measurement of respiratory enzyme activity

The activity of mitochondrial respiratory complexes I (NADH dehydrogenase), II (succinate dehydrogenase), and III (cytochrome *c* reductase) was assayed as described previously^28^. Briefly, to estimate the activity of complexes I + III, NADH and cytochrome *c* (oxidized form) were used as substrates, and the reduction of cytochrome *c* was monitored by measuring absorbance at a wavelength of 550 nm. To estimate the activity of complexes II + III, sodium succinate and cytochrome *c* (oxidized form) were used as substrates, and the reduction of cytochrome *c* was monitored as described above. For the estimation of complex IV (cytochrome c oxidase) activity, cytochrome *c* (reduced form) was used as a substrate, and the oxidation of cytochrome c was measured at 550 nm.

### Western blot analysis

Proteins were separated by SDS-PAGE in 10% gels and transferred to polyvinylidene difluoride (PVDF) membranes. Membranes were blocked with PVDF Blocking Reagent for Can Get Signal (Toyobo, Osaka, Japan) for 1 h. The membranes were incubated with primary antibodies against mouse GCAT (1:1,000; sc-86466, Santa Cruz Biotechnology, Dallas, TX, USA), mouse SHMT2 (1:1,000; #12762, Cell Signaling Technology, Danvers, MA, USA), β-ACTIN (1:10,000; A1978, Sigma, St. Louis, MO, USA) or α-TUBULIN (1:50,000; T5168, Sigma, St. Louis, MO, USA) for 1 h at room temperature; Can Get Signal Immunoreaction Enhancer Solution 1 (Toyobo) was used for dilution. The membranes were then incubated with horseradish peroxidase-conjugated secondary antibodies against goat IgG (1:20,000; HAF109, R&D Systems, Minneapolis, MN, USA), rabbit IgG (1:10,000; G-21234, Thermo Fisher Scientific, Waltham, MA,USA) or mouse IgG (1:10,000; G-21040, Life Technologies, Carlsbad, CA, USA) for 1 h at room temperature; Can Get Signal Immunoreaction Enhancer Solution 2 (Toyobo) was used for dilution. Bands were detected with a bio-imaging analyser, EZ-Capture ST (ATTO, Tokyo, Japan) using ECL Select Western Blotting Detection Reagent (GE Healthcare, Buckinghamshire, UK).

### Statistical analysis

Data were analysed by Student’s *t*-test or one-way ANOVA followed by Tukey’s multiple comparison test. *P* values of less than 0.05 were considered significant.

### Data availability

All data generated or analysed during this study are included in this published article and its Supplementary Information files.

## Electronic supplementary material

Supplemental figure 1
