# Supplementary material for: Mice deficient in the Shmt2 gene have mitochondrial respiration defects and are embryonic lethal
Source: Sci Rep. 2018 Jan 11;8:425. doi: 10.1038/s41598-017-18828-3 (PMC5765156; doi:10.1038/s41598-017-18828-3)
Supplement: Supplementary file 1 — Supplemental figure 1 [file 41598_2017_18828_MOESM1_ESM.pdf]

Mice deficient in the *Shmt2* gene have mitochondrial respiration defects and are embryonic lethal

Haruna Tani[1,†], Sakiko Ohnishi[1,†], Hiroshi Shitara[1,2,†], Takayuki Mito[3], Midori Yamaguchi[2], Hiromichi Yonekawa[2], Osamu Hashizume[3], Kaori Ishikawa[1,3], Kazuto Nakada[1,3], Jun-Ichi Hayashi[4,*]

[1]Graduate School of Life and Environmental Sciences, University of Tsukuba, 1-1-1 Tennodai, Tsukuba, Ibaraki 305-8572, Japan.

[2]Laboratory for Transgenic Technology, Tokyo Metropolitan Institute of Medical Science, 2-1-6 Kamikitazawa, Setagaya-ku, Tokyo 156-8506, Japan.

[3]Faculty of Life and Environmental Sciences, University of Tsukuba, 1-1-1 Tennodai, Tsukuba, Ibaraki 305-8572, Japan.

[4]University of Tsukuba, 1-1-1 Tennodai, Tsukuba, Ibaraki 305-8572, Japan.

[†]These authors contributed equally to this work.

[*]Correspondence and requests for materials should be addressed J.-I. H. (Tel: +81 29 853 6650; Fax: +81 29 853 6614; E-mail: jih45@biol.tsukuba.ac.jp)

Figure S1

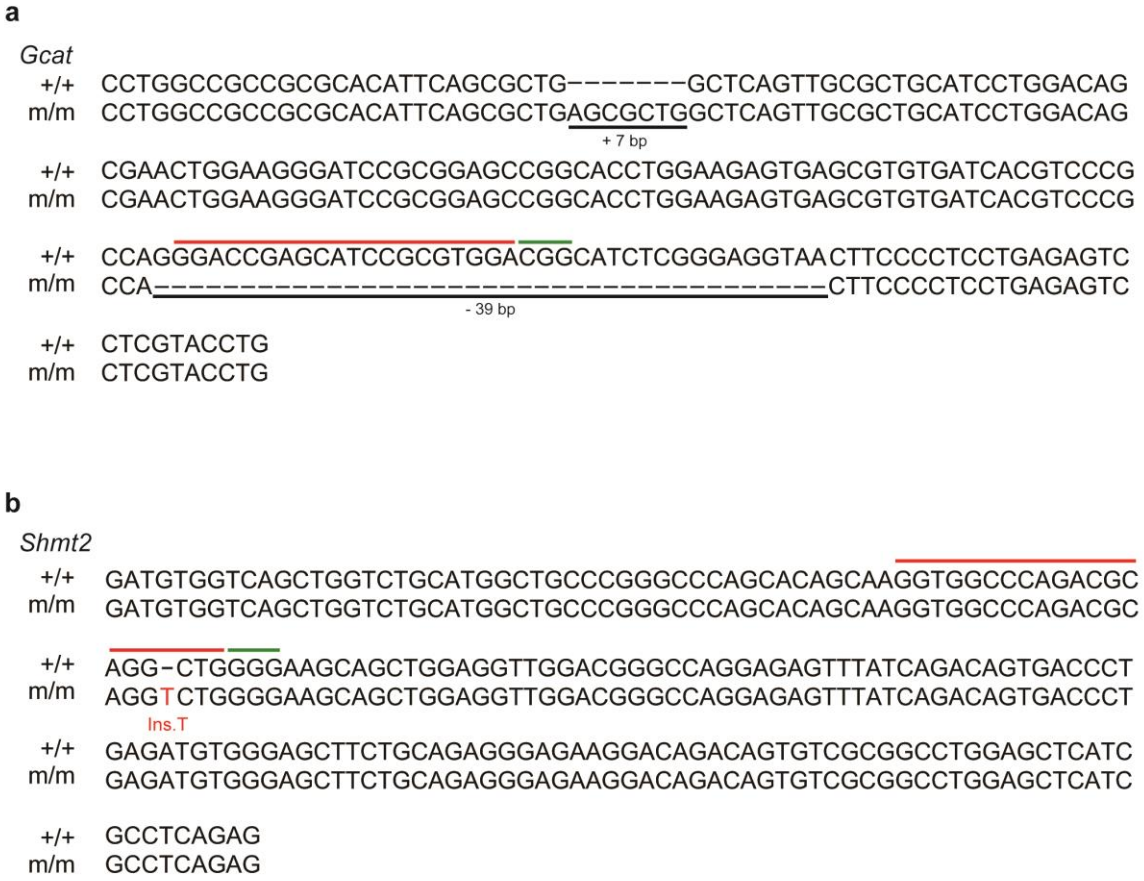

Figure S1. Confirmation of the mutations in *Gcat* (**a**) and *Shmt2* (**b**) by sequence analysis. **a** The target region of the *Gcat* gene in *Gcat* m/m mice. Red line, target sequence; green line, protospacer-adjacent motif (PAM) sequence; +7 bp, an insertion; −39bp, a deletion. **b** The target region of the *Shmt2* gene in MEFs from an m/m embryo. Red line, target sequence; green line, PAM sequence; ins. T, a T insertion.
